# Impact of Heat Stress on Gene Expression in the Hypothalamic–Pituitary–Ovarian Axis of Hu Sheep

**DOI:** 10.3390/ani15152189

**Published:** 2025-07-25

**Authors:** Jianwei Zou, Lili Wei, Yishan Liang, Juhong Zou, Pengfei Cheng, Zhihua Mo, Wenyue Sun, Yirong Wei, Jun Lu, Wenman Li, Yulong Shen, Xiaoyan Deng, Yanna Huang, Qinyang Jiang

**Affiliations:** 1 College of Animal Science and Technology, Guangxi University, Nanning 530004, China; 2218401009@st.gxu.edu.cn (J.Z.); 15977285951@163.com (L.W.); liangys2025@163.com (Y.L.); chengpengfei98@163.com (P.C.); 19978391494@163.com (Z.M.); zqaswy@163.com (W.S.); gxuwyr@163.com (Y.W.); 2218391030@st.gxu.edu.cn (J.L.); liwenman010215@163.com (W.L.); 15207759234@163.com (Y.S.); 18376025735@163.com (X.D.); 2 Guangxi Key Laboratory of Animal Breeding, Disease Control and Prevention, Guangxi University, Nanning 530004, China

**Keywords:** heat stress, hypothalamic–pituitary–ovarian (HPO) axis, gene expression, reproductive dysfunction, Hu sheep

## Abstract

Heat stress (HS) is a major environmental factor negatively impacting the reproductive performance of livestock. This study investigates the molecular mechanisms of heat stress on the hypothalamic–pituitary–ovarian (HPO) axis in Hu sheep. A heat-stressed animal model was established, and high-throughput RNA sequencing (RNA-seq) was employed to analyze gene expression in the hypothalamus, pituitary, and ovarian tissues of both control and heat-stressed groups. The results revealed significant changes in estrus behavior, hormone secretion, and reproductive health in heat-stressed sheep, with a shortened estrus duration, prolonged estrous cycles, and decreased levels of FSH, LH, E_2_, and P4. A total of 520, 649, and 482 differentially expressed genes (DEGs) were identified in the hypothalamus, pituitary, and ovary, respectively. The DEGs were enriched in pathways related to hormone secretion, neurotransmission, cell proliferation, and immune response, with significant involvement of the p53 and cAMP signaling pathways. Tissue-specific responses to heat stress were observed, with distinct regulatory roles in each organ, including GPCR activity and cytokine signaling in the hypothalamus, calcium-regulated exocytosis in the pituitary, and cilium assembly and ATP binding in the ovary. Key genes such as *SYN3*, *RPH3A*, and *IGFBP2* were identified as central to the coordinated regulation of the HPO axis. These findings provide new insights into the molecular basis of heat stress-induced impairments in reproductive function—manifested by altered estrous behavior, reduced hormone secretion (FSH, LH, E_2_, and P4), and disrupted gene expression in the hypothalamic–pituitary–ovarian (HPO) axis—and offer potential targets for improving heat tolerance and reproductive regulation in sheep.

## Simple Summary

Heat stress (HS) significantly affects the reproductive performance of Hu sheep by disrupting estrus behavior, hormone secretion, and follicular development. This study established a heat stress model in Hu sheep and used high-throughput RNA sequencing (RNA-seq) to analyze the molecular responses of the hypothalamic–pituitary–ovarian (HPO) axis. Results showed that heat stress shortened estrus duration, prolonged estrus cycles, and decreased reproductive hormone levels (FSH, LH, E_2_, P4). Transcriptomic analysis identified 520, 649, and 482 differentially expressed genes (DEGs) in the hypothalamus, pituitary, and ovary, respectively, involved in hormone secretion, neurotransmission, cell proliferation, and immune response. Key genes like *SYN3*, *RPH3A*, and *IGFBP2* were found to regulate the HPO axis. This study offers new insights into the molecular mechanisms of heat stress-induced reproductive dysfunction and provides potential targets for improving heat tolerance and reproductive regulation in sheep.

## 1. Introduction

With the ongoing progression of global climate change, heat stress (HS) has become a critical environmental factor that adversely affects the health, productivity, and reproductive capacity of livestock and poultry species [1,2,3]. Hu sheep, one of China’s prominent indigenous meat sheep breeds, are well recognized for their strong adaptability, rapid growth, and high reproductive efficiency [4]. Featuring a superior meat quality and remarkable prolificacy, Hu ewes exhibit high annual lambing rates and serve as a core breed for meat sheep production in Southern China [5,6]. In sheep and other small ruminants, elevated ambient temperatures can suppress estrous behavior, reduce conception rates, and impair follicular development and ovarian function [7,8,9], thereby posing a significant threat to the economic returns and sustainable development of the livestock industry.

The hypothalamic–pituitary–ovarian (HPO) axis is a central neuroendocrine pathway that regulates reproductive function in mammals [10]. This axis regulates follicular development, ovulation, and the synthesis of sex hormones such as estrogen and progesterone, thereby maintaining the normal reproductive cycle and fertility in female animals [11,12]. Any disruption to any component of the HPO axis by environmental changes may trigger a cascade of effects, leading to hormonal imbalance and reproductive dysfunction [13].

In recent years, increasing attention has been given to the effects of heat stress on the reproductive system of livestock, with most studies focusing on hormonal fluctuations, changes in physiological behavior, and molecular mechanisms within individual tissues [14,15]. Research into ruminants such as cattle and sheep has demonstrated that heat stress significantly accelerates physiological responses including respiratory rate and heart rate, reduces circulating reproductive hormone levels, disrupts endocrine balance, impairs follicular development, and severely compromises reproductive health [16,17,18]. Moreover, with the rapid advancement of transcriptomic technologies, numerous heat stress-related genes and regulatory pathways have been identified in species such as cattle and sheep. For instance, in heat-stressed animals, the expression of immune-related genes such as *NFKB1*, *IL1B*, and *IL10* was found to be upregulated, while genes involved in fatty acid metabolism such as *ACOT7* and *CPT1C* were also increased, and *RAC1* and *PRKAB2* expression was downregulated [19,20]. However, most current studies have focused on single tissues, and there is a lack of a systematic investigation into the overall response mechanisms of the hypothalamic–pituitary–ovarian (HPO) axis. In particular, the coordinated molecular regulation among the hypothalamus, pituitary, and ovary under heat stress conditions remains largely unclear.

In this study, Hu sheep were used as the experimental model to investigate the effects of heat stress. By establishing a heat-stressed animal model, we performed high-throughput RNA sequencing (RNA-seq) to conduct a comprehensive transcriptomic analysis of the hypothalamus, pituitary, and ovarian tissues from both control and heat-stressed groups. Differentially expressed genes (DEGs) were systematically identified, followed by Gene Ontology (GO) annotation, Kyoto Encyclopedia of Genes and Genomes (KEGG) pathway enrichment, and protein–protein interaction (PPI) network analysis to uncover the molecular responses and key regulatory factors of the HPO axis under heat stress. This study provides new insights into the molecular mechanisms underlying heat stress-induced reproductive impairment in sheep and offers a theoretical basis and potential targets for the future breeding of heat-tolerant lines and reproductive regulation.

## 2. Materials and Methods

### 2.1. Animals and Experimental Diets

Twelve healthy 2- to 3-year-old multiparous Hu ewes (41.69 ± 1.17 kg) were randomly selected from Guangxi Anxin Animal Husbandry Co., Ltd. in Dahua County, Guangxi, China. All the ewes were non-pregnant multiparous females that had just completed an estrous cycle prior to the start of the experiment. The animals were randomly assigned to either a control group (Con, *n* = 6) or a heat-stressed group (HS, *n* = 6) and housed in two separate artificial climate-controlled rooms equipped with air conditioners and heaters. The control group room was maintained at 23 °C, while the HS group room was kept at 38 °C from 08:00 to 18:00 and at 28 °C from 18:00 to 08:00 the next day. To ensure consistent heat stress exposure among individuals, all sheep were trimmed every three weeks. The experiment lasted for 68 days. Dry-bulb temperature and humidity were recorded every 30 min using an automatic temperature and humidity recorder (Jingchuang Electronics, Xuzhou, China) suspended 1.7 m above the ground. The Temperature–Humidity Index (THI) was calculated using the following formula: THI = (1.8 × Tdb + 32) − (0.55 − 0.0055 × RH)(1.8 × Tdb − 26.8) [21], where Tdb is the dry-bulb temperature (°C), and RH is the relative humidity (%). During the experiment, both groups were fed the same diet and had ad libitum access to water. The composition and nutritional values of the diet are shown in Table 1. Estrus detection was performed daily for at least 30 min using teaser rams wearing estrus detection cloths. Ewes showing active approach behavior, tail wagging, and willingness to mate were considered to be in estrus.

### 2.2. Sample Collection

At the end of the experiment, blood samples were collected from the jugular vein of all the Hu ewes (*n* = 12). Estrus detection was performed daily throughout the entire experimental period, and all samples were collected 12 h after the onset of estrus to ensure consistency in reproductive status at the time of sampling. Serum was separated by centrifugation at 3000× *g* for 15 min at 4 °C and stored at −80 °C for further analysis. Additionally, according to the Chinese agricultural standard NY/T 3469-2019 [22], three 2- to 3-year-old multiparous Hu ewes (*n* = 6) from each group were randomly selected for slaughter 12 h after estrus onset. Hypothalamic, pituitary, and ovarian tissues were rapidly frozen in liquid nitrogen for RNA-seq analysis.

### 2.3. Serum Biochemical Level Analysis

Serum concentrations of reproductive hormones, including follicle-stimulating hormone (FSH), luteinizing hormone (LH), estradiol (E_2_), and progesterone (P4), were measured in 12 Hu sheep using ELISA kits from Quanzhou Jiubang Biotechnology Co., Ltd. (Quanzhou, Fujian, China), following the manufacturer’s instructions. The assay sensitivities and intra-/inter-assay coefficients of variation (CVs) were FSH: 0.1 mIU/mL; intra-CV: 5.0%; inter-CV: 7.5%LH: 0.1 mIU/mL; intra-CV: 4.5%; inter-CV: 6.0%E_2_: 1.0 pg/mL; intra-CV: 6.5%; inter-CV: 8.0%P4: 0.05 ng/mL; intra-CV: 5.5%; inter-CV: 7.0%.

### 2.4. RNA Extraction, cDNA Library Construction, and Sequencing

Total RNA was extracted from ovarian tissues using TRIzol reagent (Invitrogen Life Technologies, Carlsbad, CA, USA) according to the manufacturer’s instructions. RNA concentration and purity were measured using a NanoDrop spectrophotometer (Thermo Scientific, Waltham, MA, USA). RNA purity and integrity were further assessed using a NanoPhotometer^®^ (IMPLEN, Westlake Village, CA, USA) and the RNA Nano 6000 Assay Kit on the Agilent Bioanalyzer 2100 system (Agilent Technologies Inc., City of Santa Clara, CA, USA). According to the manufacturer’s recommendations, 1 μg of total RNA per sample was used as input material to generate sequencing libraries using the NEBNext^®^ Ultra™ II RNA Library Prep Kit for Illumina (New England Biolabs Inc., Ipswich, MA, USA). Library quality was assessed using the Agilent 2100 Bioanalyzer (Agilent Technologies Inc., City of Santa Clara, CA, USA) with the Agilent High Sensitivity DNA Kit (Agilent Technologies Inc., City of Santa Clara, CA, USA; 5067-4626). Paired-end 150 bp (PE150) sequencing was performed on the Illumina NovaSeq platform (Illumina Inc., San Diego, CA, USA).

### 2.5. Quality Control and Transcriptome Assembly

Raw FASTQ-format data were first processed using FastQC (v0.22.0) to assess the sequencing quality. Adapter sequences were trimmed from the 3′ ends, and low-quality reads with an average score below Q20 were removed. The resulting clean reads were aligned to the sheep reference genome (GCF_016772045.2_ARS-UI_Ramb_v3.0) using HISAT2 (v2.1.0). The reference genome index was also built with HISAT2. SAMtools was used to convert SAM files into BAM format. The mapped reads were then assembled into transcripts using StringTie (v2.1.7) for each sample.

### 2.6. Gene Expression Quantification and Differential Expression Analysis

HTSeq (v0.9.1) was used to count the number of reads mapped to each gene, representing the raw expression level. Gene expression levels were normalized using FPKM (Fragments Per Kilobase of transcript per Million mapped reads) or TPM (Transcripts Per Million). Differential gene expression analysis between comparison groups was performed using DESeq2 (v1.38.3). Genes were considered differentially expressed if they met the criteria |log2FoldChange| > 1 and *p*-value < 0.05.

### 2.7. GO and KEGG Gene Enrichment, Functional Analysis, and Gene Interaction Analysis

GO functional enrichment analysis and Kyoto Encyclopedia of Genes and Genomes (KEGG) pathway enrichment analysis of DEGs from the hypothalamus, pituitary, and ovary tissues of Hu sheep were performed using clusterProfiler software (v4.6.0). *p*-values were calculated using the hypergeometric distribution method, with a significance threshold set at *p*-value < 0.05. Interactions among differentially expressed genes were explored using the STRING database (http://string-db.org/, accessed on 15 May 2024), and the resulting interaction networks were visualized using Cytoscape 3.2.1.

### 2.8. Protein–Protein Interaction (PPI) Network Analysis

The lists of differentially expressed genes identified in each group were imported into the STRING database (v 12.0) (https://cn.string-db.org/, accessed on 15 May 2024). The interaction confidence score threshold was set to 0.4. The exported protein interaction networks were then visualized and analyzed using Cytoscape software.

### 2.9. RT-qPCR Validation of RNA-Seq Data

Total RNA was extracted and purified from tissues using RNAiso Reagent (Vazyme, Nanjing, China). cDNA synthesis was performed using the PrimeScript™ RT reagent Kit with gDNA Eraser (TaKaRa, Dalian, China) according to the manufacturer’s instructions. Primer pairs were designed using NCBI Primer-BLAST and synthesized by Genis Biotechnology (Genis, Nanning, China) (see Supplementary Table S1). Real-time quantitative PCR (RT-qPCR) was conducted using the TB Green^®^ Premix Ex Taq™ II kit (TaKaRa, Dalian, China) on a CFX−96 Real-Time PCR Detection System (Bio-Rad, Hercules, CA, USA). The 20 μL reaction system contained 10 μL TB Green Premix Ex Taq™ II (TaKaRa, Dalian, China), 0.8 μL each of forward and reverse primers (10 μmol/L), 2 μL DNA template, and 6.4 μL RNase-free ddH_2_O. The amplification conditions were as follows: initial denaturation at 95 °C for 30 s, followed by 45 cycles of 95 °C for 5 s and 60 °C for 30 s. The relative expression level of each gene was calculated using the 2^−ΔΔCt^ method, normalized to GAPDH expression.

### 2.10. Statistical Analysis

All statistical analyses were performed using R software (v4.2.2) and GraphPad Prism (v9.0). Group comparisons were conducted using independent-sample t-tests, with results expressed as mean ± SEM. Significance was set at *p* < 0.05. For transcriptomic data, differential expression analysis was conducted using DESeq2, and genes with an adjusted *p* < 0.05 and |log_2_FC| > 1 were considered significantly differentially expressed. Gene Ontology (GO) and KEGG pathway enrichment analyses of DEGs were performed using the clusterProfiler package based on the hypergeometric distribution. Hierarchical clustering of DEGs was conducted using Euclidean distance and the average linkage method. Pearson correlation analysis was used to assess inter-sample consistency. A protein–protein interaction (PPI) network of DEGs was constructed using the STRING database, and hub genes were identified based on degree centrality.

## 3. Results

### 3.1. The Effects of Heat Stress on Estrous Behavior in Hu Sheep

**Figure 1:**
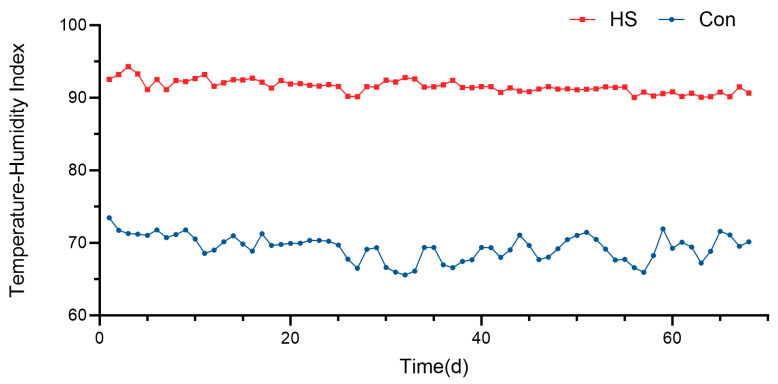
Average Temperature–Humidity Index (THI) of the climate-controlled rooms for the control and heat-stressed Hu sheep.

Studies have shown that a Temperature–Humidity Index (THI) below 72 indicates no heat stress (HS), 72 ≤ THI < 78 indicates mild HS, 78 ≤ THI < 90 indicates moderate HS, and THI ≥ 90 indicates severe HS [21]. As shown in Table 2, during the entire experimental period, the average THI in the housing of the control group Hu sheep was 69.36 ± 1.9, while that of the HS group was 91.54 ± 0.93. The THI in the housing of the HS group was significantly higher than that of the control group (Figure 1, *p* < 0.05). Moreover, compared with the control group, the HS group showed a significantly prolonged estrous cycle (17.85 ± 0.65 vs. 16.37 ± 0.42 d, *p* < 0.05) and a significantly shortened estrus duration (32.22 ± 0.77 vs. 34.67 ± 0.67 h, *p* < 0.05).

### 3.2. Effects of Heat Stress on Serum Concentrations of FSH, LH, E_2_, and P4 in Hu Sheep

As shown in Table 3, the serum concentrations of follicle-stimulating hormone (FSH), luteinizing hormone (LH), estradiol (E_2_), and progesterone (P4) in the heat-stressed (HS) group were significantly lower than those in the control group (*p* < 0.05). Specifically, FSH decreased from 13.07 ± 1.30 in the control group to 10.46 ± 1.19 mIU/mL in the HS group, and LH dropped from 30.21 ± 5.06 to 24.69 ± 3.09 mIU/mL. E_2_ levels declined from 213.20 ± 20.81 to 166.27 ± 11.58 pg/mL, and P4 levels decreased from 26.61 ± 2.08 to 20.24 ± 2.36 ng/mL. The E_2_/P4 ratio increased from 8.10 ± 1.59 in the control group to 8.27 ± 0.76 in the HS group (*p* > 0.05).

### 3.3. RNA-Seq Data Quality and Gene Expression Analysis

RNA-seq analysis was conducted on hypothalamus, pituitary, and ovarian tissues from both control and heat-stressed Hu sheep. As shown in Table 4, A a total of 18 cDNA libraries were sequenced using the Illumina NovaSeq platform, yielding 925,643,296 clean reads. Of these, 902,313,944 reads were successfully mapped to the reference genome. The GC content ranged from 45.00 to 47.18%, and the Q30 values ranged from 95.38 to 96.42%, indicating that the sequencing data were of high quality and suitable for subsequent analyses.

### 3.4. Identification and Analysis of Differentially Expressed Genes in the Hypothalamic Tissue of Hu Sheep

**Figure 2:**
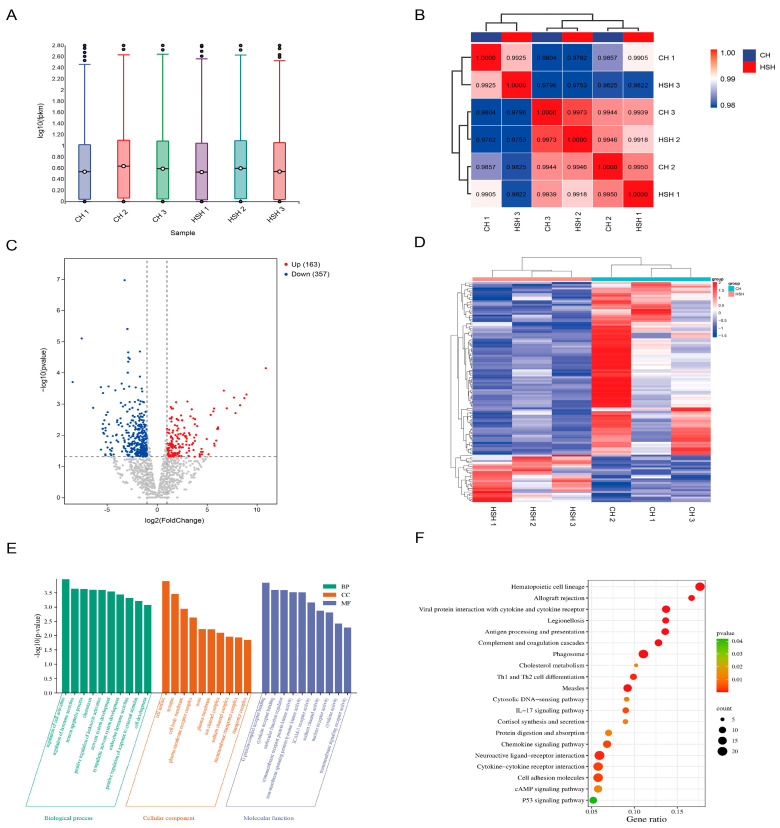
Identification and bioinformatic analysis of differentially expressed genes (DEGs) in hypothalamic tissue. (**A**) Boxplot showing gene expression levels (log10 FPKM) for each sample. (**B**) Correlation analysis between samples. (**C**) Heatmap of DEG expression in hypothalamic tissues. (**D**) Volcano plot of DEGs in hypothalamic tissue. (**E**) GO enrichment analysis of DEGs in hypothalamic tissue. (**F**) KEGG enrichment analysis of DEGs in hypothalamic tissue.

The expression levels of genes in the hypothalamus were quantified using FPKM (Fragments Per Kilobase of transcript per Million mapped reads). Statistical analysis of FPKM values showed consistent gene expression distributions within hypothalamic tissues of both the CH and HSH groups (Figure 2A). A high degree of correlation was observed among individual hypothalamic samples (correlation coefficients ranged from 0.9 to 1, *p* < 0.05) (Figure 2B). A total of 520 differentially expressed genes (DEGs) were identified between the CH and HSH groups, including 163 upregulated and 357 downregulated transcripts (Figure 2C, Supplementary Table S2). These DEGs exhibited distinct expression clustering patterns between the two groups (Figure 2D). Systematic GO and KEGG enrichment analyses were performed on the DEGs in the hypothalamus. GO analysis identified 1188 significantly enriched biological terms (*p* < 0.05), including “regulation of hormone secretion,” “sodium channel complex,” “G protein-coupled receptor binding,” and “cytokine receptor binding” (Figure 2E). KEGG analysis revealed that the DEGs were significantly enriched in the p53 signaling pathway and cAMP signaling pathway (Figure 2F).

### 3.5. Identification and Analysis of Differentially Expressed Genes in the Pituitary Tissue of Hu Sheep

**Figure 3:**
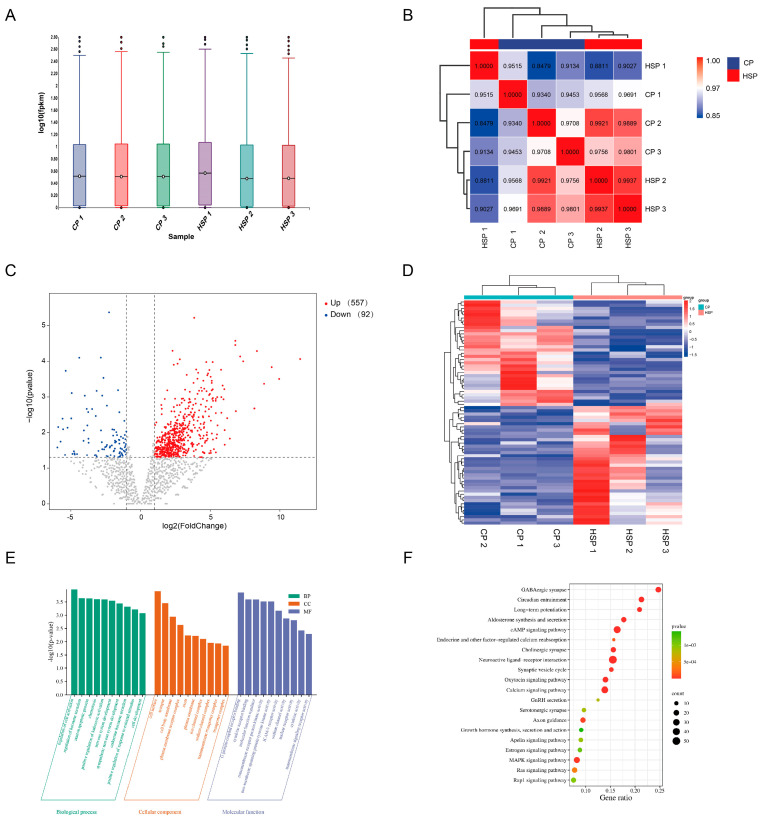
Identification and bioinformatic analysis of differentially expressed genes (DEGs) in pituitary tissue. (**A**) Boxplot showing gene expression levels (log10 FPKM) for each sample. (**B**) Correlation analysis between groups. (**C**) Heatmap of DEG expression in pituitary tissues for each sample. (**D**) Volcano plot of DEGs in pituitary tissue. (**E**) GO enrichment analysis of DEGs in pituitary tissue. (**F**) KEGG enrichment analysis of DEGs in pituitary tissue.

The expression levels of genes in the pituitary were quantified using FPKM (Fragments Per Kilobase of transcript per Million mapped reads). Statistical analysis of FPKM values indicated consistent gene expression distributions within pituitary tissues of both the CP and HSP groups (Figure 3A). A high degree of correlation was observed among individual pituitary samples, with correlation coefficients ranging from 0.8 to 1 (*p* < 0.05, Figure 3B). A total of 649 differentially expressed genes (DEGs) were identified between the CP and HSP groups, including 557 upregulated and 92 downregulated transcripts (Figure 3C, Supplementary Table S3). These DEGs exhibited distinct expression clustering patterns between the two groups (Figure 3D). Systematic GO and KEGG enrichment analyses were conducted for the DEGs in the pituitary. GO analysis identified 1092 significantly enriched biological terms, including “calcium ion-regulated exocytosis of neurotransmitter,” “excitatory synapse,” “calmodulin binding,” and “calcium-release channel activity” (Figure 3E, *p* < 0.05). KEGG analysis revealed that DEGs were significantly enriched in the calcium signaling pathway and the cAMP signaling pathway (Figure 3F, *p* < 0.05).

### 3.6. Identification and Analysis of Differentially Expressed Genes in the Ovarian Tissue of Hu Sheep

**Figure 4:**
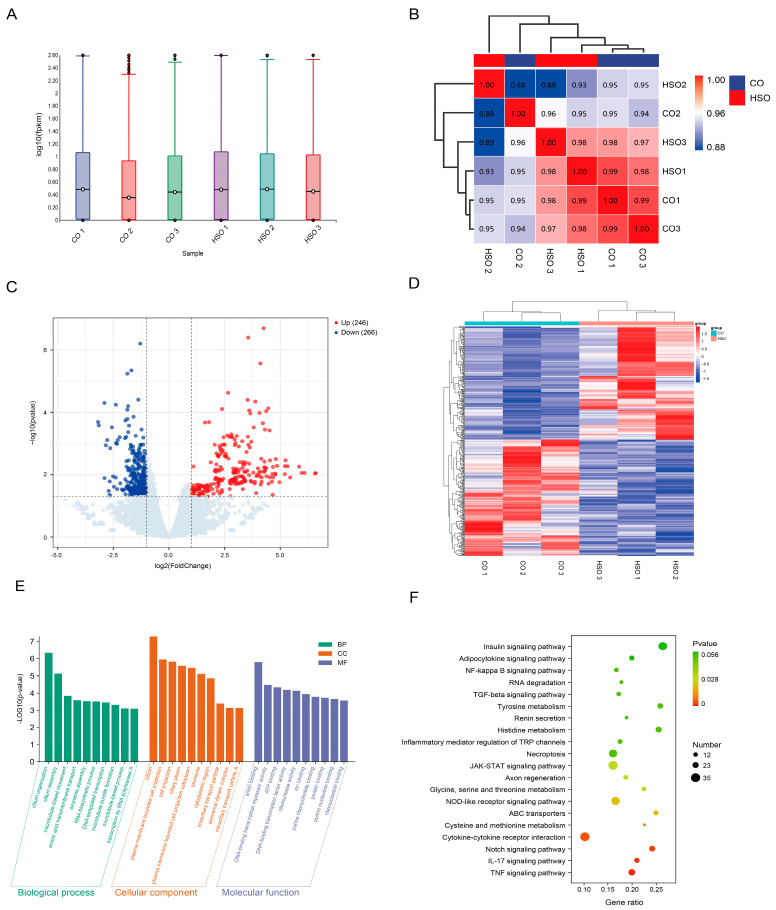
Identification and bioinformatic analysis of differentially expressed genes (DEGs) in ovarian tissue. (**A**) Boxplot showing gene expression levels (log10 FPKM) for each sample. (**B**) Correlation analysis between groups. (**C**) Heatmap of DEG expression in ovarian tissues for each sample. (**D**) Volcano plot of DEGs in ovarian tissue. (**E**) GO enrichment analysis of DEGs in ovarian tissue. (**F**) KEGG enrichment analysis of DEGs in ovarian tissue.

The expression levels of genes in the ovary were quantified using FPKM (Fragments Per Kilobase of transcript per Million mapped reads). Statistical analysis of FPKM values showed consistent gene expression distributions within ovarian tissues of both the CO and HSO groups (Figure 4A). A high degree of correlation was observed among individual ovarian samples, with correlation coefficients ranging from 0.8 to 1 (*p* < 0.05, Figure 4B). A total of 510 differentially expressed genes (DEGs) were identified between the CO and HSO groups, including 241 upregulated and 269 downregulated transcripts (Figure 4C, Supplementary Table S4). These DEGs exhibited distinct expression clustering patterns between the two groups (Figure 4D). Systematic GO and KEGG enrichment analyses were performed on the DEGs in the ovary. GO analysis identified 1151 significantly enriched biological terms (*p* < 0.05), including “cilium organization,” “cilium,” “anion binding,” and “ATP binding” (Figure 4E). KEGG analysis revealed that the DEGs were significantly enriched in the p53 signaling pathway and the cAMP signaling pathway (Figure 4F, *p* < 0.05).

### 3.7. Clustering Analysis of Differentially Expressed Genes in the Hypothalamic–Pituitary–Ovarian (HPO) Axis

**Figure 5:**
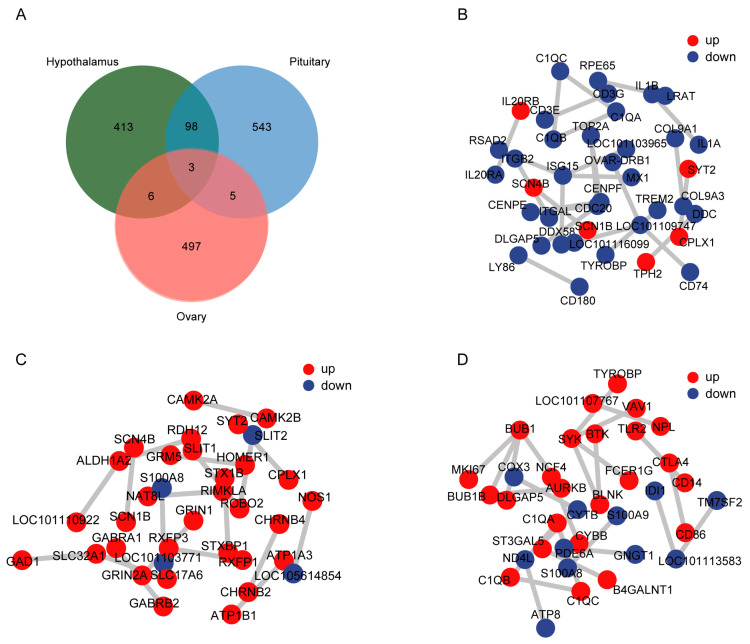
Inter-tissue correlation analysis of differentially expressed genes in the hypothalamic–pituitary–ovarian (HPO) axis. (**A**) Venn diagram of significantly differentially expressed genes in the hypothalamus, pituitary, and ovary. (**B**) Gene interaction network formed by differentially expressed genes in hypothalamic tissue. (**C**) Gene interaction network formed by differentially expressed genes in pituitary tissue. (**D**) Gene interaction network formed by differentially expressed genes in ovarian tissue.

The results showed that compared to the control group, 520, 649, and 482 genes were significantly differentially expressed in the hypothalamus, pituitary, and ovarian tissues of heat-stressed Hu sheep, respectively (Figure 5A). A total of 101 common DEGs were identified between the hypothalamus and pituitary, primarily related to nervous system development and neurotransmission, such as *SAMSN1*, *GABRR2*, and *GABRR1*. Nine common DEGs were identified between the hypothalamus and ovary, mainly associated with immune response and cell proliferation, including *IFIH1*, *CDC20*, and *GEM*. Eight common DEGs were identified between the pituitary and ovary, linked to neurotransmission and metabolic processes, such as *SYNPR*, *NSF*, and *GCNT4*. Notably, *SYN3*, *RPH3A*, and *IGFBP2* were significantly differentially expressed in all three tissues. These genes regulate synaptic signaling, neuroendocrine activity, and steroid hormone biosynthesis. The DEGs in the hypothalamic–pituitary–ovarian axis were significantly enriched in the protein–protein interaction (PPI) network *(p* < 0.05), with *ISG15*, *ALDH1A2*, *C1QB*, and *BUB1* identified as key hub genes (Figure 5B–C).

### 3.8. RT-qPCR Validation of circRNAs

**Figure 6:**
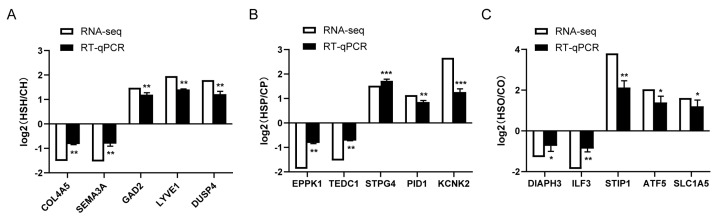
Validation of RNA-Seq data by RT-qPCR for DEGs in hypothalamic, pituitary, and ovarian tissues. (**A**) RT-qPCR validation analysis in hypothalamic tissue. (**B**) RT-qPCR validation analysis in pituitary tissue. (**C**) RT-qPCR validation analysis in ovarian tissue. * *p* < 0.05, ** *p* < 0.01, *** *p*< 0.001.

To validate our RNA-seq data, five DEGs were selected from each of the hypothalamus, pituitary, and ovarian tissues of Hu sheep for RT-qPCR analysis (Figure 6A–C). The results confirmed that the DEGs exhibited consistent expression trends between the RT-qPCR and RNA-seq, thereby verifying the accuracy of the sequencing data.

## 4. Discussion

Heat stress is a major environmental factor that disrupts reproductive performance in ruminants, leading to reduced estrous behavior, lower conception rates, and impaired ovarian function [23,24,25,26]. Beyond endocrine interference, recent studies suggest that heat stress also affects reproduction via altered gene expression and transcriptional regulation [27,28,29]. The hypothalamic–pituitary–ovarian (HPO) axis plays a central role in regulating female reproductive function [30]. Based on prior findings, we hypothesized that heat stress alters gene expression along the HPO axis and disrupts hormone secretion and estrous behavior in Hu sheep. To test this, we established a heat stress model in Hu sheep, monitored estrous behavior, measured serum reproductive hormones, and performed RNA-seq on hypothalamic, pituitary, and ovarian tissues. Results showed that heat stress significantly shortened estrus duration, prolonged the estrous cycle, and reduced serum FSH, LH, E_2_, and P4 levels, while increasing the E_2_/P4 ratio. Transcriptome analysis revealed 520, 649, and 482 differentially expressed genes (DEGs) in the hypothalamus, pituitary, and ovary, respectively, indicating widespread disruption of neuroendocrine and reproductive signaling. These findings demonstrate that heat stress impairs reproductive function by reducing hormone levels and disturbing endocrine coordination, offering new insights into the molecular basis of heat stress-induced reproductive dysfunction in Hu sheep.

It has been reported that when the Temperature–Humidity Index (THI) exceeds 88 units, clear signs of heat stress (HS) can be observed in animals [31]. Prolonged exposure to high temperatures significantly alters estrous behavior and endocrine hormone levels in ruminants [32,33]. To minimize hormonal fluctuations caused by differences in reproductive cycles, all samples in this study were collected 12 h after the onset of estrus in all Hu ewes. The results showed that the levels of FSH, LH, estradiol (E_2_), and progesterone (P4) in the heat-stressed group were significantly lower than those in the control group, which is consistent with previous research findings [34,35]. These changes suggest that heat stress impairs the function of the hypothalamic–pituitary–ovarian (HPO) axis, thereby affecting the timely secretion of reproductive hormones and the normal regulation of the estrous cycle. In addition, FSH and LH are essential hormones for follicular development and ovulation, while E_2_ and P4 are involved in endometrial preparation and the maintenance of pregnancy. Heat stress can suppress ovarian follicular activity and impair luteal function, leading to the reduced secretion of reproductive hormones [36,37]. In this study, the heat-stressed group showed significantly decreased levels of FSH, LH, estradiol (E_2_), and progesterone (P4), which is consistent with previous findings. Notably, although both E_2_ and P4 levels declined, the reduction in P4 was more pronounced, resulting in a slight increase in the E_2_/P4 ratio. This may indicate insufficient luteal support and an imbalance between estrogen and progesterone signaling, with the underlying mechanism likely closely related to dysfunction of the hypothalamic–pituitary–ovarian (HPO) axis.

Further analysis revealed that the DEGs were mainly enriched in hormone secretion, neurotransmission, cell proliferation, and immune response pathways—hallmarks of physiological disruption under heat stress, consistent with previous studies [38,39,40]. The p53 and cAMP signaling pathways, known regulators of reproductive hormone signaling, follicular development, and steroidogenesis [41,42,43,44], were significantly affected in our GO and KEGG analyses, suggesting that heat stress may impair Hu sheep’s fertility by inducing cell cycle arrest, apoptosis, and hormonal dysregulation. Interestingly, tissue-specific responses were observed. In the hypothalamus, DEGs were mainly linked to G protein-coupled receptor (GPCR) activity and cytokine signaling, essential for neuroendocrine regulation and stress adaptation [45,46,47]. In the pituitary, enriched pathways included calcium-regulated exocytosis and synaptic transmission, key for hormonal signals’ reception and secretion [48,49]. In the ovary, DEGs were associated with cilium assembly and ATP binding, implying roles in follicular development and steroid hormone synthesis [50,51], potentially reflecting direct effects of heat stress on follicle maturation and oocyte quality.

This study also revealed a number of common DEGs across different tissues, suggesting a coordinated regulatory mechanism of the HPO axis under heat stress. For example, 101 shared DEGs were identified between the hypothalamus and pituitary, including genes such as *SAMSN1* and *GABRR2*, which are closely related to synaptic transmission and neurogenesis [52,53]. Between the hypothalamus and ovary, shared genes such as *CDC20* and *GEM* are mainly involved in immune regulation and cell proliferation [54,55]. Between the pituitary and ovary, genes such as *NSF* and *GCNT4* were identified, which are associated with neurotransmission and metabolic processes [56,57]. In addition, *SYN3*, *RPH3A*, and *IGFBP2* were significantly differentially expressed in all three tissues, playing essential roles in synaptic signaling regulation, neuroendocrine activity, and steroid hormone synthesis [58,59,60].

Protein–protein interaction (PPI) network analysis further revealed that differentially expressed genes (DEGs) within the HPO axis were significantly enriched in the PPI network (*p* < 0.05), and several key hub genes were identified, including Interferon-Stimulated Gene 15 (*ISG15*), Aldehyde Dehydrogenase 1 Family Member A2 (*ALDH1A2*), Complement C1q B Chain (*C1QB*), and *BUB1* Mitotic Checkpoint Serine/Threonine Kinase (*BUB1*). These genes are known to be closely associated with cell cycle regulation, immune responses, and oxidative stress [61,62,63,64], and their dysregulation may play a critical role in mediating reproductive dysfunction under heat stress. In addition, the RT-qPCR validation of selected DEGs from each tissue showed consistent expression trends with RNA-seq data, further confirming the reliability of the transcriptomic results.

## 5. Conclusions

In summary, this study systematically elucidated the impact of heat stress on the HPO axis of Hu sheep, demonstrating that heat exposure leads to consistent disruptions in estrous behavior, reproductive hormone secretion, and tissue-specific gene expression profiles in the hypothalamus, pituitary, and ovary. The differentially expressed genes (DEGs) were broadly enriched in key pathways related to neural signaling, cell cycle, immune response, and steroidogenesis, exhibiting distinct tissue-specific expression patterns. Several core genes commonly identified across multiple tissues (e.g., *SYN3*, *RPH3A*, *IGFBP2*) may play central roles in the coordinated regulation of the HPO axis. These findings provide new evidence for understanding the molecular mechanisms underlying heat stress-induced reproductive impairment and offer theoretical support and potential targets for the heat-tolerant breeding and reproductive regulation in sheep.

## Figures and Tables

**Table 1:** Dietary formula composition and nutritional level.

Ingredients (%)	Content	Nutrition Level	Content
Soybean meal	5	Dry Matter (DM)/%	58.52
Silage corn	48	Digestible Energy (DE)/(MJ/Kg)	15.18
Peanut vines	11	Crude Protein (CP)/%	8.88
Rice bran	8.3	Crude Fat (EE)/%	1.54
Dried cassava distillers’ grains	7.7	Crude Fiber (CF)/%	8.98
Premix ^1^	20	Crude Ash (Ash)/%	6.43
Total	100	Calcium (Ca)/%	0.28
		Total Phosphorus (TP)/%	0.19

Note: All values are expressed on an air-dry (as-fed) basis. ^1^ The premix contains Vitamin A 700,000 IU/kg, Vitamin E 900,000 IU/kg, Pantothenic acid 80 mg/kg, Biotin 4.0 mg/kg, Niacin 80 mg/kg, Iron (Fe) 500 mg/kg, Copper (Cu) 100 mg/kg, Manganese (Mn) 600 mg/kg, Zinc (Zn) 500 mg/kg, Iodine (I) 10 mg/kg, Selenium (Se) 2.25 mg/kg, Cobalt (Co) 2.25 mg/kg, Calcium (Ca) 12%, and Phosphorus (P) 2%.

**Table 2:** Effects of heat stress on estrus duration and estrous cycle in Hu sheep.

Items	Con Group (*n* = 6)	HS Group (*n* = 6)
THI	69.36 ± 1.9 ^b^	91.54 ± 0.93 ^a^
Duration of estrus (h)	34.67 ± 0.67 ^a^	32.22 ± 0.77 ^b^
Estrous cycle (d)	16.37 ± 0.42 ^b^	17.85 ± 0.65 ^a^

Note: Different lowercase superscript letters indicate significant differences between the Con and HS groups at the *p* = 0.05 level.

**Table 3:** Effects of heat stress on serum reproductive hormones in Hu sheep.

Items	Con Group (*n* = 6)	HS Group (*n* = 6)
FSH (mIU/mL)	13.07 ± 1.30 ^a^	10.46 ± 1.19 ^b^
LH (mIU/mL)	30.21 ± 5.06 ^a^	24.69 ± 3.09 ^b^
E2 (pg/mL)	213.20 ± 20.81 ^a^	166.27 ± 11.58 ^b^
P4 (ng/mL)	26.61 ± 2.08 ^a^	20.24 ± 2.36 ^b^
E2/P4	8.10 ± 1.59 ^a^	8.27 ± 0.76 ^a^

Note: Different lowercase superscript letters indicate significant differences between the Con and HS groups at the *p* = 0.05 level.

**Table 4:** Basic statistics of RNA sequencing data.

Items	Total Reads	GC Content (%)	Q30 (%)	Total Mapped	Multiple Mapped	Unique Mapped
CH1	57,775,932	46.20	96	56,964,559 (98.60%)	3,778,821 (6.63%)	53,185,738 (93.37%)
CH2	63,269,708	46.05	96.06	62,056,418 (98.08%)	3,967,798 (6.39%)	58,088,620 (93.61%)
CH3	64,539,062	46.10	96	63,441,668 (98.30%)	3,340,356 (5.27%)	60,101,312 (94.73%)
CP1	51,070,098	45.20	95.76	50,166,417 (98.23%)	2,553,151 (5.09%)	47,613,266 (94.91%)
CP2	54,744,876	45.03	96.09	53,744,246 (98.17%)	2,882,450 (5.36%)	50,861,796 (94.64%)
CP3	57,134,276	45.00	95.78	56,147,003 (98.27%)	2,713,457 (4.83%)	53,433,546 (95.17%)
CO1	35,823,368	47.10	95.59	34,176,209 (97.32%)	1,322,043 (3.87%)	32,854,166 (96.13%)
CO2	46,093,358	47.09	95.92	43,798,054 (96.78%)	1,695,457 (3.87%)	42,102,597 (96.13%)
CO3	41,919,740	47.04	95.38	39,918,406 (97.30%)	1,551,716 (3.89%)	38,366,690 (96.11%)
HSH1	47,258,932	46.30	95.98	46,584,008 (98.57%)	2,330,024 (5.00%)	44,253,984 (95.00%)
HSH2	51,609,568	46.25	95.95	50,761,427 (98.36%)	2,424,292 (4.78%)	48,337,135 (95.22%)
HSH3	45,713,232	46.30	95.87	44,890,635 (98.20%)	2,928,147 (6.52%)	41,962,488 (93.48%)
HSP1	67,615,950	45.60	95.94	66,528,725 (98.39%)	2,935,594 (4.41%)	63,593,131 (95.59%)
HSP2	54,728,132	45.02	96.42	53,781,581 (98.27%)	2,772,163 (5.15%)	51,009,418 (94.85%)
HSP3	49,742,860	45.80	95.9	48,857,721 (98.22%)	2,705,473 (5.54%)	46,152,248 (94.46%)
HSO1	46,099,642	47.18	95.72	44,053,758 (97.55%)	1,603,493 (3.64%)	42,450,265 (96.36%)
HSO2	43,421,104	47.00	95.54	41,442,144 (97.53%)	1,498,265 (3.62%)	39,943,879 (96.38%)
HSO3	47,083,458	46.94	95.52	45,000,965 (97.55%)	1,663,015 (3.70%)	43,337,950 (96.30%)

Note: CH, CP, and CO denote control group hypothalamus, pituitary and ovary samples, while HSH, HSP, and HSO represent those from the heat-stressed group.

## Data Availability

The original contributions presented in the study are included in the article; further inquiries can be directed to the corresponding author.

## Supplementary Materials

The following supporting information can be downloaded at https://www.mdpi.com/article/10.3390/ani15152189/s1, Table S1: Gene-specific primer pairs used for RT-qPCR; Table S2: Differentially expressed genes between CH and HSH groups; Table S3: Differentially expressed genes between CP and HSP groups; Table S4: Differentially expressed genes between CO and HSO groups.

## Abbreviations

The following abbreviations are used in this manuscript:

HS	Heat stress
FSH	Follicle-stimulating hormone
LH	Luteinizing hormone
E_2_	Estradiol
P_4_	Progesterone
HPO	Hypothalamic–pituitary–ovarian
SYN3	Synapsin III
RPH3A	Rabphilin 3A
IGFBP2	Insulin-like Growth Factor Binding Protein 2
ISG15	Interferon-Stimulated Gene 15
ALDH1A2	Aldehyde Dehydrogenase 1 Family Member A2
C1QB	Complement C1q B Chain
BUB1	BUB1 Mitotic Checkpoint Serine/Threonine Kinase
