# Supplementary material for: Impact of Heat Stress on Gene Expression in the Hypothalamic–Pituitary–Ovarian Axis of Hu Sheep
Source: Animals (Basel). 2025 Jul 25;15(15):2189. doi: 10.3390/ani15152189 (PMC12345516; doi:10.3390/ani15152189)
Supplement: Supplementary file 1 [file animals-15-02189-s001.zip › Supplementary Table S1.pdf]

Supplementary Table S1. Gene-specific primer pairs used for RT-qPCR.

Genes	Sense primer (5'→3')	Antisense primer (5'→3')	Product length (bp)
EPPK1	CTGGACCAAGACACGTACCC	TCTCGGTGACTCCTCTCCTG	119
TEDC1	GTCTTCTGGCAGTGGATGCT	GATACCAGGGACGCACAGAG	91
STPG4	CTGCCTGTTCAAAAACCCCG	CCATTTTGGCTATGGTGCGG	84
PID1	GTCAGGCTGCACAGAAAAGC	CACACTTGGAACGGTCGGAT	111
KCNK2	GCTGTGTACTCTTCGTCGCT	TAGTCGCCAAAGCCAATGGT	118
COL4A5	CCAATGAGCATGGAACCCCT	GGTCTGACTGTGGACTGCAA	105
SEMA3A	GTAGGCAAAGGCCAGGACAT	GCCCTCTCAAATTCGTGGGT	103
GAD2	GTATGGAGCCTTTGACCCCC	ATCAGTAATCCCCCACCCCA	99
LYVE1	TGCAGAATCATGGGGGTCAC	TTCAACCTGGTCTTGGCTGG	120
ATRNL1	ACAGTGGCTTCCTGGTGAAC	TTTCGCAGACAAGCCCATCT	100
DIAPH3	ACAATATCAACTGCAGCGCAA	CATCACAGGCCTCCTTCTCAA	114
ILF3	TGGTGCTGCTGTGTAAGGAG	TCTGTGACAGCAGTGAGCTG	88
STIP1	AAGAGCTGGGGAATGAAGCC	TTTCGAAGTACACGGCTGCT	129
ATF5	TGGGCTGGCTCGTAGACTAT	CCGTTCGGTCATCCAGTCAG	149
SLC1A5	ATCTTACCGCTGGTGGTGTG	CAAGGAAAAAGAGCAGCGCC	100
GAPDH	TCTCAAGGGCATTCTAGGCTAC	GCCGAATTCATTGTCGTACCAG	151
